# What is a segment?

**DOI:** 10.1186/2041-9139-4-35

**Published:** 2013-12-17

**Authors:** Roberta L Hannibal, Nipam H Patel

**Affiliations:** 1 Departments of Molecular and Cell Biology and Integrative Biology, University of California, 519A LSA #3200, Berkeley, CA 94720-3200, USA; 2 Present Address: Department of Genetics, Stanford University School of Medicine, Stanford, CA 94305, USA

**Keywords:** Evolution, Metamere, Pseudosegment, Segmentation

## Abstract

Animals have been described as segmented for more than 2,000 years, yet a precise definition of segmentation remains elusive. Here we give the history of the definition of segmentation, followed by a discussion on current controversies in defining a segment. While there is a general consensus that segmentation involves the repetition of units along the anterior-posterior (a-p) axis, long-running debates exist over whether a segment can be composed of only one tissue layer, whether the most anterior region of the arthropod head is considered segmented, and whether and how the vertebrate head is segmented. Additionally, we discuss whether a segment can be composed of a single cell in a column of cells, or a single row of cells within a grid of cells. We suggest that ‘segmentation’ be used in its more general sense, the repetition of units with a-p polarity along the a-p axis, to prevent artificial classification of animals. We further suggest that this general definition be combined with an exact description of what is being studied, as well as a clearly stated hypothesis concerning the specific nature of the potential homology of structures. These suggestions should facilitate dialogue among scientists who study vastly differing segmental structures.

## Why is the definition of segmentation important?

‘The only dogmatic statement we are justified in making is, that when a region exhibits during development a sufficient number of the essential structures of a typical segment, it may be assumed to be at true metamere. What is “sufficient” has to be decided in each case.’ ES Goodrich, 1897 [1]

‘It is difficult to find a [concept] in the whole of zoology that is so vaguely defined, but, at the same time, so universally employed as…metamerism.’ RB Clark, 1964 [2]

**Figure 1:**
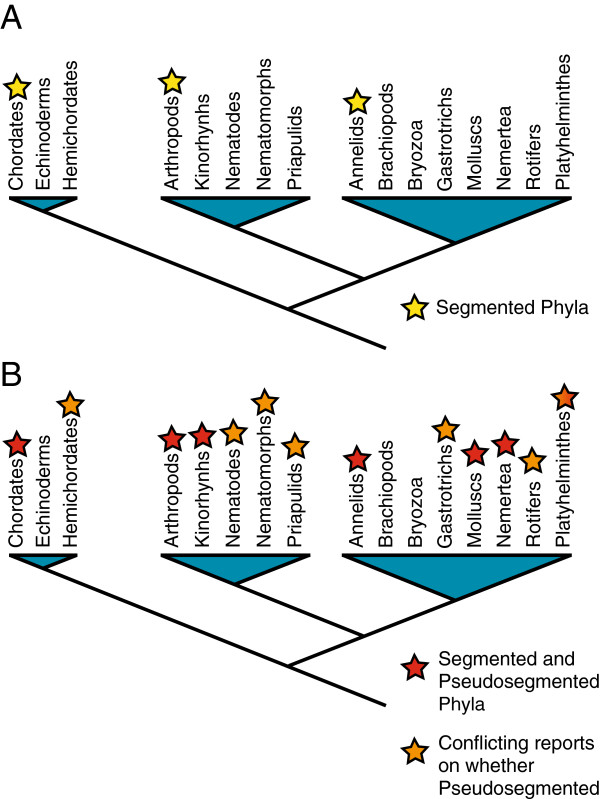
**Phylogenetic relationship among segmented and unsegmented phyla.** Phylogeny of bilatarians based on [14]; segmented and pseudosegmented animals identified after [7,12,13]. **(A)** Segmented phyla (yellow stars) are more closely related to unsegmented phyla than to each other. **(B)** Segmentation is no longer a rare characteristic if both segmented and pseudosegmented phyla are considered (red stars mark groups identified as segmented or pseudosegmented in several papers, orange stars mark groups identified as segmented or pseudosegmented in one paper and unsegmented in another). Here, ‘pseudosegmented’ is meant solely to distinguish traditionally segmented chordates, arthropods and annelids from other phyla with repetition of units with anterior-posterior polarity along the anterior-posterior axis. It does not necessarily mean that there is a biological distinction between these groups based on their repeated units.

Arthropods, annelids, and chordates are some of the most successful and diverse animal groups, as defined by species number and anatomical complexity. The success of these groups may be due to their segmented trunk region, which may enhance locomotion and feeding [2-4]. While these groups share the trait of trunk segmentation, it is controversial whether segmentation is homologous, since these groups are more closely related to unsegmented phyla than to each other (Figure 1A) [5-11]. If segmentation is difficult to evolve, the most parsimonious explanation for three unrelated segmented groups would be that segmentation evolved once, but was subsequently lost in all taxa related to the arthropods, annelids, and vertebrates. In this case, we would expect to find evidence of loss of segmentation in related groups. Conversely, if segmentation is easy to gain, we would expect to find multiple unrelated segmented taxa. In fact, there may be such evidence in modern and fossil taxa, as there are a number of groups besides the arthropods, annelids, and vertebrates that display serially repeated units, and could therefore be considered segmented (Figure 1B) [7,12,13]. However, in order to use these groups to infer the evolutionary history of segmentation, we must first resolve whether these taxa are actually segmented. The main obstacle in resolving this issue is that there is no precise definition of segmentation. Instead, there is a range of definitions, depending on what animals and what parts of these animals were studied by a particular author. Here, we give an overview of the various definitions of segmentation, and we discuss current controversies in defining a segment.

## The history of the definition of segmentation

The Greeks first recorded the observation that some animals are made of segments, reiterated units along the anterior-posterior (a-p) axis. Aristotle [15] named and classified a group of animals as ‘insects’ , because of their segmental nature. Entomon (‘insect’ in Greek) is derived from the Greek word *entomos*, meaning ‘to cut up’ , and was used because these animals had ‘nicks’ or ‘cuts’ on their back or bellies, corresponding to boundaries between segments. Latin and related modern languages continued this theme, as the English word ‘insect’ is derived from the Latin *insecure*, which also means ‘to cut up’.

The scientific revolution of the seventeenth century brought science to the forefront of Western society and set the stage for renewed interest in segmentation in the nineteenth century. During the 1800s, Cuvier grouped arthropods and annelids into the now defunct taxon *Articulata* because of their similar segmental morphologies [16]. Goodrich also considered arthropod and annelid segments homologous. From studies on arthropods and annelids, Goodrich defined a segment as a unit, marked off from the rest of the body by transverse grooves, containing a mesodermal hollow space (coelom), a pair of nephridia (excretory glands), and a pair of ventral ganglia [1]. Goodrich also noted that, in polychaetes and arthropods, a segment also contains a pair of appendages. Besides these morphological characteristics, Goodrich used a developmental characteristic, the sequential addition of segments from anterior to posterior, to define segmentation.

Goodrich’s definition does not accurately describe all segments. As Goodrich acknowledged, segments containing all of the above criteria are rarely found, although some features can transiently be seen during development [1]. Also, Goodrich’s definition excludes segmentation in the arthropod *Drosophila* and in the chordates. In *Drosophila*, segments are formed by simultaneously subdividing the entire body, in contrast with Goodrich’s developmental requirement of adding segments from the posterior end [1,17]. In chordates, segments are added progressively from the posterior, but they do not have a number of Goodrich’s other morphological characteristics [18]. Another caveat to Goodrich’s definition is that if the formation of reiterated structures is linked, then using all of them to define a segment would be no more informative then using one of them [19]. For example, segmentation of the coelom, nephridia, and ganglia might all be based on the same molecular pattern. Then, since all three traits would be a read-out of the same pattern, any or all of them could be used equivocally to define that segmental pattern.

Around the same time as Goodrich, Bateson defined segmentation as a ‘more-or-less’ coincident repetition of elements from many organ systems along the a-p body axis [20]. Unlike Goodrich, however, Bateson did not define what these elements had to be, and based his definition on trunk segmentation in vertebrates, as well as segmentation in arthropods and annelids. While Bateson’s definition is applicable to trunk segmentation in the arthropods, annelids and chordates, many scientists prefer a more precise definition.

While segments can be thought of as the repetition of a variety of structures, Clark suggested that reiteration of coelomic sacs and accompanying muscle was the defining characteristic of a segment [2,3]. The coelom is a fluid-filled body cavity derived from the mesoderm. Clark suggested that segmentation involving the coelom and muscles has evolved because of the need for better locomotion, as a large, unsegmented, coelomic sac would have impeded movement. Clark proposed that the division of the coelom facilitates movement by allowing the body to bend at the regions between compartments and that this division of the coelom is accompanied by muscle segmentation.

Clark’s theory could potentially explain the advantage of segmentation in the arthropods, annelids, and chordates, since segmentation could have evolved for improved locomotion in all of these groups. However, segmentation does not seem to be correlated with any kind of locomotion. Clark himself acknowledged that ribbon-like animals swim in the same manner whether they are segmented or not, although he suggested that extant animals might not be good representatives of the ancestors of segmented phyla [2]. Like swimming, burrowing does not correlate with segmentation, since many burrowing worms are not segmented [13,21].

Another caveat to Clark’s theory is DuPorte’s [22] argument that, ‘There is no real foundation for the belief in a fundamental relation between coelomic sacs and metamerism.’ DuPorte suggests that coelomic sacs may be a stage in mesoderm differentiation and therefore does not have a direct relationship to segmentation. He based this on the observation that protostome mesoderm originates as a solid mass, but only differentiates into coelomic sacs after this mass has already been divided into segments. Clark’s definition of segmentation has also been used sparingly in modern times, perhaps because of the emergence of the arthropod ectoderm as a model for segmentation. Experimental evidence suggests that, in arthropods, the ectoderm can segment normally without the mesoderm [23-27].

Analysis of modern molecular data has also failed to produce a concise definition of segmentation. Instead of finding a few genes and mechanisms that could be used to define segmentation, studies have yielded a large number [28]. Even when homologous genes or gene families are involved in segmentation, they often play different roles in different animals [8,11,29]. For example, although members of the Notch, Wnt, and fibroblast growth factor signaling pathways oscillate to produce segmental trunk mesoderm in mouse, chick, and zebrafish, the individual genes that oscillate differ between species [29]. This may be only a minor difference, in that it might not matter which component of the pathway oscillates, as long as some components oscillate [10]. Major differences are also found, especially among different species of arthropods. Within arthropods, there are multiple changes in which genes appear to be involved in segmentation, and, unlike many other arthropods and vertebrates, *Drosophila* does not have any evidence of Notch or other oscillators (for example, see [30-33]). Moreover, morphologically similar segments can be formed by different developmental and molecular mechanisms in the same animal. For example, although all of the somites, segmental units in vertebrate trunk mesoderm, appear morphologically homologous in zebrafish, there is variation in how they form, depending on their position along the a-p axis [34]. The development of the anterior trunk, posterior trunk, and tail somites depend on different genes or have different degrees of dependence on the same genes, or both. Instead of yielding a precise definition of a segment, modern molecular studies have highlighted the complexity of segmentation.

## Current controversies in the field of segmentation

While there is a general agreement that segmentation involves reiterated units along the a-p body axis, there are still a number of points of contention. The major debates surrounding the definition of a segment are: (1) whether a segment can be composed of only one tissue layer, (2) whether the anterior arthropod head is considered segmented, and (3) whether and how the vertebrate head is segmented. An additional complicating factor for defining segmentation is whether a segment can be composed of a single cell in an a-p column of cells, or a single row of cells within a grid of cells. We will discuss this issue first, as it has bearing on the contentions over segmentation in the literature.

### Can a segment be a single cell in a column of cells?

**Figure 2:**
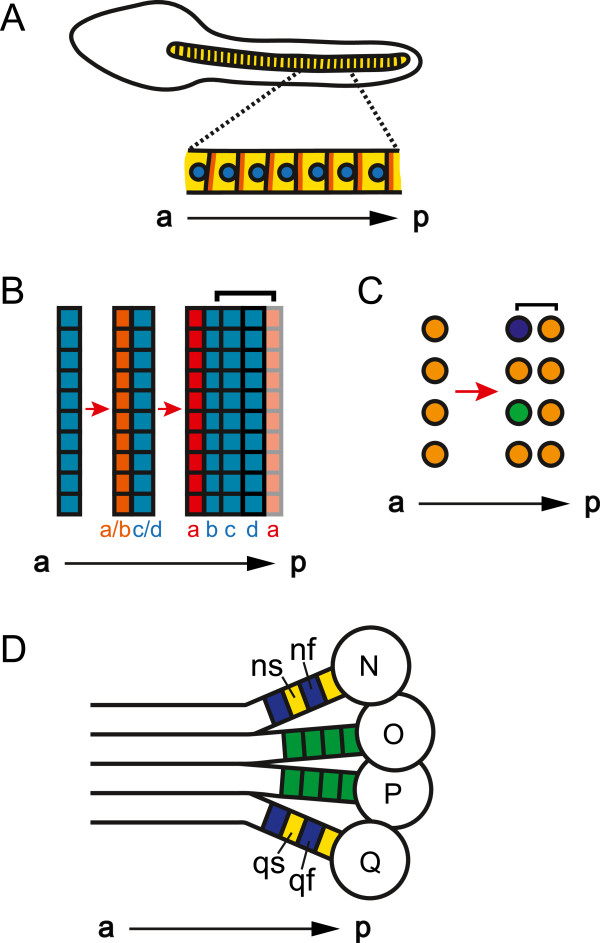
**A single cell (row) may be a segment. (A)** Lateral view of a *Ciona savignyi* late tailbud-stage embryo where the notochord is composed of a column of single cells (yellow). The planar-cell polarity proteins Prickle and Strabismus (orange) are located at the anterior and the nucleus (blue) is located at the posterior of each cell. (**A** after [35].) **(B, C)** Segmentation in the trunk of *Parhyale hawaiensis*. Left side of the embryo is depicted, right side is mirror image. Red arrows represent progression in time. **(B)** Once ectodermal cells condense into rows (PSPRs), each PSPR divides to produce one parasegment of ectoderm. After the first PSPRs division, *Ph*-*hedgehog* (*Ph-hh*, orange) is expressed in the anterior row (row a/b) [40]. After the second division, both En and *Ph*-*hh* (red) are expressed in the anterior row (row a) [36]. While the division of one PSPR produces one parasegment of ectoderm, in general, one segment’s worth of ectoderm (bracket) forms from the Engrailed (En) negative cells of one parasegment (rows b to d), and the En positive cells from another parasegment (row a) [36]. **(C)** One row of mesoblasts produces one segment’s worth of mesoderm (bracket). After the first mesoblasts division, *Ph-twist* (green) and *Ph-even*-*skipped* (purple) are expressed in a subset of the anterior daughters [27,37,40]. **(D)** Segmentation in the leech ectoderm. One side of the embryo is depicted, other side is mirror image. The ectoderm is formed from the progeny of four ectoteloblasts, N, O, P, and Q, [41]. Each progeny, or blast cell (green), of O and P gives rise to one segmental unit. However, two adjacently produced blasts cells from N and Q, ns (yellow) and nf (blue) and qs (yellow) and qf (blue), respectively, give rise to one segmental unit. a, anterior; p, posterior, PSPR, parasegment precursor row.

**Figure 3:**
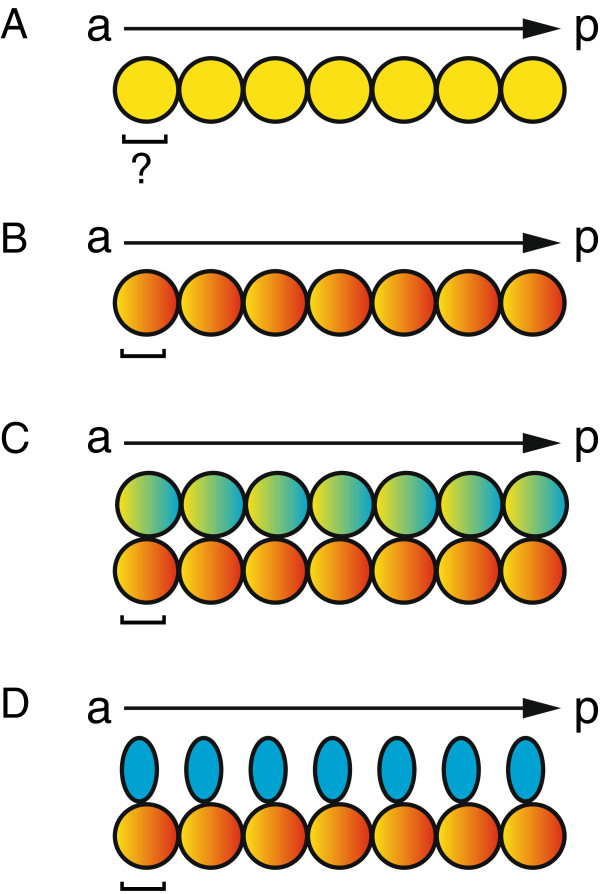
**A segment can be composed of one or more tissue layers.** Bracket marks one cell or segment, except in **(A)**, where it marks one putative cell or segment. **(A)** Yellow circles represent cells (putative segments) in one layer of tissue. **(B)** Each cell in a column of cells can be called a segment if there is a-p cell polarity in each cell. Cells (circles) now have a-p cell polarity, represented by difference in coloration from yellow (anterior) to red (posterior). **(C, D)** Segments are often defined as having reiterated units (segments) composed of derivatives of both mesoderm (yellow-red circles) and ectoderm (yellow-blue **(C)** and blue **(D)** circles). Each dorsal-ventral row of cells forms one segment. **(C)** Both the mesoderm and the ectoderm have a-p cell polarity, represented by difference in coloration. **(D)** Only the mesoderm (yellow-red) has intrinsic a-p cell polarity. The ectoderm (blue), does not have reiterated pattern on its own, but does contribute to the segmental pattern of each segment as a whole, since it is associated with the anterior of each segment. a, anterior; p, posterior.

During development, some animals have an arrangement of cells along the a-p axis in which each single cell (or row of single cells) could be considered a segment. For example, the notochord of the sea squirt *Ciona savignyi* is composed of a single column of cells (Figure 2A) [35]. Similarly, the trunk of the arthropod *Parhyale hawaiensis*, as well as the trunks of other malacostracan crustaceans, is composed of columns of cells where segments arise from single-cell-wide rows within a grid of cells (Figure 2B,C) [36-38]. For a column of cells along the a-p axis to be considered a column of segments, each cell or row of cells needs a definable anterior and posterior (Figure 3A,B) [39]. Having an anterior and a posterior distinguishes each cell from its neighbors, while making each cell a reiteration of a unit. In segments composed of two or more rows of cells, an a-p segmental pattern can be accomplished by having different morphology or gene expression in anterior versus posterior rows of a single segment (segment polarity). In segments composed of only a single cell, or that are only a single cell wide, the anterior and posterior of the single cell must exhibit molecular or morphological a-p asymmetry (cell polarity).

The most obvious molecular mechanism to distinguish the anterior from the posterior of cells is the planar-cell polarity pathway. Indeed, expression of members of this pathway supports the hypothesis that each cell in the notochord of the sea squirt *Ciona savignyi* is a segment (Figure 2A) [35]. By expressing tagged *Ciona* Prickle and Strabismus proteins, Jiang *et al*. [35] revealed localization of these proteins at the anterior edge of each notochord cell. Additionally, they found that each notochord nucleus is asymmetrically positioned near the posterior of each cell. These data show that each *Ciona* notochord cell has a-p cell polarity, supporting the classification of the *Ciona* notochord as segmented, with the segmental units being single cells along the a-p axis.

Unlike *Ciona*, a-p cell polarity has not yet been found in the trunk of the malacostracan arthropod *Parhyale hawaiensis* during the stage where segments, or more accurately for the ectoderm, parasegments, are destined to form from single-cell-wide rows of cells within a grid of cells. *Parhyale* segments are composed of both ectoderm and mesoderm. In the trunk, ectodermal parasegments form via the division of parasegment precursor rows (PSPRs), while mesodermal segments form via the division of mesoblasts, which are produced via the asymmetrical division of mesodermal stem cells (mesoteloblasts; Figure 2B,C) [36,37]. In *Parhyale*, each parasegment/segment has polarity after the first division of the PSPRs and mesoblasts, as there is differential gene expression in the anterior versus posterior row of each segment (Figure 2B,C) [27,37,40]. It will be interesting to determine whether a-p polarity is established before PSPR and mesoblast division, as well as what molecular pathways govern this patterning. As both cell types divide along the a-p axis, there may be intrinsic asymmetric determinants, and therefore a-p cell polarity, in the PSPRs and mesoblasts. Alternatively, the PSPRs and mesoblasts may only have intrinsic instructions for dividing in an a-p orientation, and then require subsequent signaling from the anterior of the embryo to distinguish the anterior daughter from the posterior daughter.

The discovery of molecular markers of a-p polarity in *Parhyale* segmental precursor cells will also resolve the question of whether segmental precursor cells are segments if they do not divide. If the ectoderm is ablated in *Parhyale*, mesoteloblasts still divide to form mesoblasts [27]. However, these mesoblasts do not divide nor do they express known markers of segment polarity normally seen after the first mesoblast division. If these mesoblasts have a-p cell polarity, they would be segments, and segmentation of the ectoderm and mesoderm could be considered to occur independently of one another. If individual mesoblasts are not considered segments because they lack a-p polarity, then the mesoderm requires either a permissive or an instructive signal from the ectoderm to gain segmental identity. An instructive signal from the ectoderm would further complicate the debate on defining a segment. If the ectoderm is required to impart a segmental pattern onto the mesoderm, then segmentation in *Parhyale* could be seen as only an ectodermal characteristic, and the general definition of segmentation would then include that the segmental pattern must be an intrinsic property of the germ layer being studied. We do not suggest using intrinsic pattern as a criteria for segmentation, as gathering this level of information would make it hard, if not impossible, to define even universally agreed upon segmental tissue as segmented.

As with malacostracan arthropods, segments in annelids can form from single-cell-wide precursors. In the leech, segments form from the asymmetrical division of teloblasts (Figure 2D) [41]. On either side of the body, progeny of four ectoteloblasts, N, O, P, and Q, and one mesoteloblast, M, come together to form a segment. Each progeny, or blast cell, of M, O, and P gives rise to one segmental unit. Although, unlike the malacostracan *Parhyale*, where a single-cell-wide row of cells gives rise to one segment/parasegment, in the leech, the progeny of each blast cell can spread over more than one segment and intermix with progeny of neighboring blast cells. Moreover, for the leech teloblasts N and Q, two adjacently produced blast cells give rise to one segmental unit. N alternatively gives rise to the blast cells ns and nf, while Q alternatively gives rise to qs and qf [41,42]. These alternate blast cells have different fates, as ns gives rise mostly to anterior neurons and epidermis, while nf gives rise to mostly posterior neurons, peripheral neurons, and neuropil glia, and qs gives rise to both ventral and dorsal cells, while qf only gives rise to dorsal cells [42]. Experiments with the more tractable N lineage support the hypothesis that ns and nf are different from birth. Ablation experiments indicate that ns and nf are not an equivalence group [42]. Additionally, molecular segment polarity is found in the progeny, as the activated form of the cell cycle protein Cdc42 is expressed in higher levels in ns versus nf [43]. These data suggest that N, and by extension, Q, produce segments with intrinsic segmental polarity. Therefore, the N and Q lineage provide a model for further studies on how teloblasts may impose intrinsic segmental polarity on their progeny. It will be interesting to explore whether N and Q use similar mechanisms as the single progeny teloblasts M, O and P, and also as teloblasts in other systems such as the malacostracans.

The vertebrate trunk axons are an excellent example of how segmental pattern can be non-intrinsic. Vertebrate trunk axons are arranged in a reiterated pattern along the a-p axis. These axons contribute to the overall segment polarity within each trunk segment by running through the anterior half of each somite [44]. However, the segmental arrangement of axons is extrinsic, caused by axon guidance cues in the anterior half of the somite. Therefore, the segmental pattern of axons is purely dependent on the polarity of the somites. These data suggest that trunk axons must be considered with the somites in order to be segmental.

### Can a segment be composed of only one tissue layer?

The arthropods, annelids, and chordates are universally considered segmented. However, there are a number of other animal groups that also display serially repeated units, and could therefore also be considered segmented (Figure 1B) [7,12,13]. To distinguish these serially repeated units from undisputed segments, these units are often called pseudosegments or metameres, depending on the author [7,12,13]. Here, we will use ‘pseudosegments’ to refer to these structures, as ‘metameres’ has often been used as a synonym for segments (for example, [1]).

While the only difference between the pseudosegmented and segmented animals may be taxonomic classification, one possible biological difference could be that the arthropods, annelids, and chordates are the only groups with segments composed of both ectodermal and mesodermal derivatives. Segments in arthropods and annelids are derived from both the ectoderm and mesoderm and have segmental pattern in both tissue layers (Figure 3C) [11,25]. In vertebrates, although trunk segments are composed of both the ectoderm-derived nervous system and the mesoderm, there is only segmental pattern in the mesoderm (Figure 3D). There is pattern in the ectoderm-derived rhombomeres of the vertebrate head, but, as there is contention about the segmental status of rhombomeres, they will be discussed in more depth in a later section.

Some pseudosegmented animals have reiteration only in the ectoderm, but there are other so-called pseudosegmented animals with reiterations in both the ectoderm and the mesoderm. The bdelloid rotifers, some species of nematodes, and chiton, a type of mollusk, only have reiteration in the ectoderm [12]. Bdelloid rotifers have repeated rings of intraepithelial skeletal laminae, some species of nematodes have repeated cuticular rings, and chitons have reiterated dorsal plates. However, other so-called pseudosegmented animals have reiterations in both the ectoderm and the mesoderm. The bodies of kinorhynchs are composed of 13 to 14 units with repetitive ganglia, muscles, and epidermal and cuticular structures [12]. Therefore, the criteria of segments being composed of both ectoderm and mesoderm is not sufficient to separate ‘classically’ segmented and so-called pseudosegmented animals. Moreover, as there are many invertebrate animals whose development and anatomy are still not well characterized, there are likely to be more species that have segmental pattern in at least one tissue layer. More data on pseudosegmented animals will also help determine whether there is an actual distinction between reiterated structures in segmented versus pseudosegmented phyla.

An evolutionary argument also suggests that requiring segments to be composed of both ectodermal and mesodermal derivatives, versus one tissue layer, is an artificial distinction [45]. According to Budd [45], instead of considering segmentation as a property of the entire animal, segmentation should be thought of as a property of an organ system. Budd defines segmentation as a characteristic of organ systems, because he views the evolution of segmentation as a gradual accumulation of reiterated organ structures. If one organ system becomes reiterated first and another system becomes reiterated later in evolution, then the distinction between having multiple reiterated structures, or segments, and having only one reiterated structure, or pseudosegments, is artificial. Therefore, any reiterated organ system should be considered segmented, and there would be no reason to consider the segmented organ systems of arthropods, annelids, and chordates as distinct from the segmented organ systems of pseudosegmented animals.

Just as segmentation could evolve in one organ system at a time, segmentation could also be secondarily lost in one organ system but not another. Evidence for secondary simplification is found in Echiura and in mollusks. Echiura is a group of marine worms that are most probably highly derived annelids [46-48]. While Echiura share many developmental traits with the annelids, they lack the epidermal and muscular segmentation of bona fide segmented worms, leading to debate over their relationship. However, recent phylogenies place them within the annelids [47,48]. Moreover, immunohistochemical analysis of neuronal markers shows that the nervous system of the Echiura *Bonellia viridis* is arranged in an organized, serial fashion, similar to the nervous systems of segmented worms [46]. These data suggests that the Echiura evolved from a segmented ancestor and later lost most segmental characteristics. As with the case of Echiura, segmentation may have been secondarily lost in some mollusks. The cephalopods are considered to be unsegmented. However, recent phylogenies group them with the Monoplacophorans, shelled deep-sea mollusks that have the segmental characteristics of serially repeated gills, nephridia, and muscles [49,50]. While these data could suggest that Monoplacophorans independently evolved segmentation, closer examination of the chambered nautilus instead suggests that segmentation may have been lost in cephalopods [51]. The nautilus has two pairs of gills, kidneys, and atria, which can be interpreted as secondary simplification from a segmented ancestor. These examples of probable secondary loss of segmentation in annelids and mollusks suggest that there is no biological distinction between segments composed of many versus one organ system, and therefore argue against the requirement that segments be composed of multiple tissue layers.

### Is the tip of the arthropod head a segment?

While most of the arthropod body is universally considered segmented, controversy exists over whether the anterior-most section of the head is a segment, and the segmental affiliation of appendage-like structures, such as the labrum [52]. As there are general reviews of these subjects elsewhere [52,53], we will focus here on the question of whether the tip of the head is a segment in the context of how phylogenetic assumptions can influence segmental classifications.

**Figure 4:**
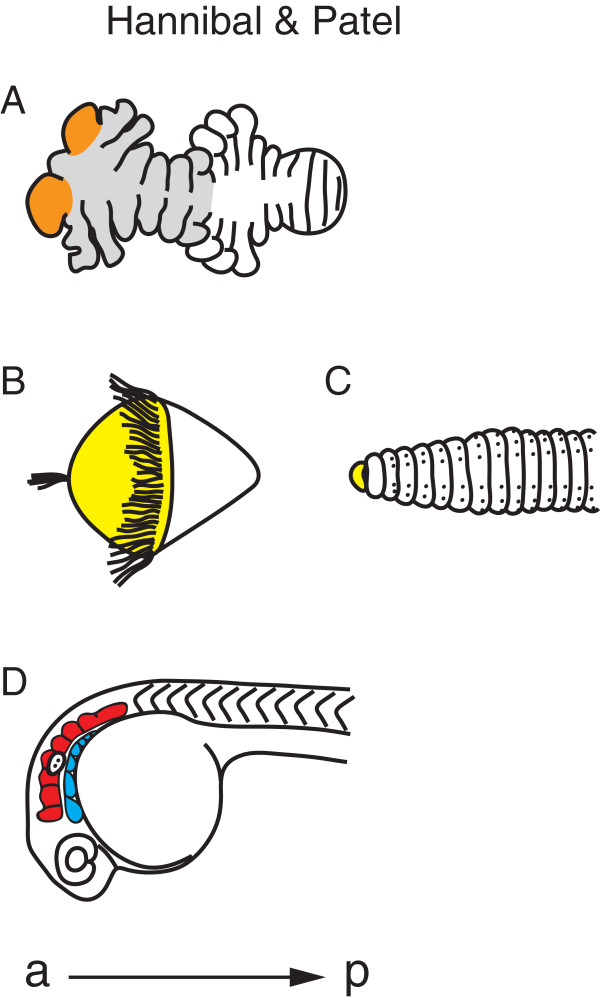
**Annelid, arthropod, and vertebrate heads. (A)** The segmental nature of the anterior region, or ocular lobe, of the arthropod head is disputed (orange). Ventral view of a 96-hour *Parhyale* embryo. Definitively segmented head segments are shaded in gray (antennae 1, antennae 2, mandibles, maxillae 1, maxillae 2, and the maxillipeds). **(B, C)** Annelids have an unsegmented anterior region, or prostomium (yellow). Lateral view of a polychaete trochophore larva **(B)**, and ventral view of the anterior region of an earthworm (**C** after [54]). **(D)** Vertebrates have head structures with segmental characteristics, such as the rhombomeres (red) and pharyngeal arches (blue). Lateral view of the anterior region of a 24-hour zebrafish embryo, head slightly curved ventrally. a, anterior; p, posterior.

The anterior-most region of the arthropod head, the ocular lobe (protocerebrum), is different from head and trunk segments, as it contains the brain and does not bear a set of antennae or other appendages (Figure 4A) [52,53]. However, the ocular lobe also has many morphological similarities to the rest of the segments, making it hard to classify as either unsegmental or segmental [22,52,53]. The difficulty in using morphology to classify the ocular lobe as a segment may have lead researchers to depend overly on phylogenetics to solve this question. Before the new molecular phylogeny, the *Articulata* hypothesis placed the annelids as close relatives to the arthropods. Therefore, it was assumed that the arthropods had an unsegmented anterior region, homologous to the unsegmented anterior region, or prostomium, of annelids (Figure 4B,C) [1,52]. The annelid prostomium lies in front of the mouth and contains the brain and sense organs. The prostomium is considered unsegmented because its embryonic origin is different from the segmented body and because it does not have characteristics of other segments, such as coelomic sacs and nephridia [52]. Additionally, in annelid species that have a trochophore larvae, the prostomium (episphere), is located anterior to the first ciliary ring (prototroch). If the annelid prostomium and the arthropod ocular lobe were homologous, then, based on annelid data, the ocular lobe would not be considered a segment. However, since the new molecular phylogeny places the arthropods and annelids in two separate megagroups of the bilatarians, the homology of the annelid prostomium and arthropod ocular lobe, and thus the unsegmented nature of the ocular lobe, has come into dispute [5].

While molecular data likely resolved the relationship between arthropods and annelids, molecular studies have not yet solved the question of whether the ocular lobe is a segment. In *Drosophila*, analysis of mutations in genes with roles in head development suggests that it is a segment [55]. Also, segmental and appendage genes are expressed in the ocular lobes of many arthropod species [36,55-61]. However, expression of segment polarity or appendage genes is limited to one, often transient, region per ocular lobe, unlike other segments, where there is a persistent domain of strong expression. This may be due to the highly derived nature of the head, or, since these genes are all pleiotropic, this may indicate a fundamental difference in the ocular lobe versus the undisputed segments of the rest of the body. While this problem may never be fully solved, studying the arthropod head will undoubtedly yield interesting insights that would never be uncovered if the assumption about head homology had not been questioned.

### What parts of the vertebrate head are segmented?

Vertebrates have structures in their heads that could be considered segmental, but that are distinct from their trunk segments. Controversy exists on whether these head structures are segmented as they do not easily fit into the already tenuous definition of segmentation formed from studies on trunk segments. Moreover, researchers have divided the vertebrate head it into segments in numerous different ways, some of which may be artificial. To clarify what parts of the head are most likely to be segmented, here we give an overview of how some head structures are probably misconstrued as segmented, followed by a brief discussion of the head structures that have the most evidence for being considered segmented, the rhombomeres and pharyngeal arches.

The vertebrate head has been divided into segments in many different ways [62-70]. While the vertebrate head probably contains a number of independently segmented structures, there is little or no reliable morphological or molecular data to support some of these claims. Two examples are the somitomeres and prosomeres. Somitomeres are defined as segmental structures of the paraxial mesoderm that resemble somites, the trunk mesodermal segments [66,67]. Somitomeres are an attractive theory, as their existence would suggest that the head and trunk share a unified segmental developmental program. However, there is only disputed scanning electron microscope data to support their existence, and no data to support a shared segmental program between the vertebrate head and trunk. Prosomeres, or forebrain segments, would also provide a framework to organize the vertebrate head [70,71]. However, while the forebrain may be partitioned, there is neither repeated morphological pattern nor molecular segmental polarity to support segmentation.

*Hox* gene expression has often been used to support the claim that the vertebrate head is segmented (for example, see [63,67]). *Hox* gene expression often correlates with anterior segmental or parasegmental boundaries and Hox proteins determine what type of structure will form from each segment [72,73]. However, this does not imply that *Hox* genes are a marker of segmentation and, therefore, *Hox* expression should not be used to define a body region, such as the head, as segmented. In support of not equating nested *Hox* gene expression to segmentation, knock-out, and overexpression of *Hox* genes alters segment identity, but do not prevent the formation of segments [72,73]. Moreover, many unsegmented animals express *Hox* genes along their a-p axis but are not segmented, no matter what definition is used (for example, see [74,75]). Although clearly important for patterning segmental structures, *Hox* gene expression by itself should not be used as molecular evidence of segmentation.

While more evidence is needed to support many of the claims for segmentation in the vertebrate head, there is morphological and molecular evidence to support two structures, the rhombomeres and pharyngeal arches, as segmented (Figure 4D) [66]. The rhombomeres are seven transient compartments in the chordate hindbrain that control neural organization and architecture [76]. If each rhombomere were a segment, we would expect a repeated pattern of segment polarity, such as the expression of a gene in only the anterior or posterior part of each rhombomere. Instead, there is a two-rhombomere periodicity of gene expression, where the *ephrin* ligands are expressed in even-numbered rhombomeres, and their receptors, the *Ephs*, are expressed in odd-numbered rhombomeres [77]. While this is often compared to the two-segment periodicity of pair-rule genes in *Drosophila*, each segment in *Drosophila* ultimately has its own segment polarity [17]. There is still polarity in each rhombomere, however, since motor neurons and their axon trajectories have a repeated pattern in each rhombomere [68].

Pharyngeal arches also have segmental characteristics. The pharyngeal arches are bulges on the lateral surface of the embryonic head that give rise to skeletal and muscular derivatives, sensory ganglia, and motor innervations [78]. The pharyngeal arches are composed from ectoderm, mesoderm, endoderm, and neural crest cells. Together, these tissues are arranged so that there is a reiterated pattern within each arch. Morphologically, cartilage is in the anterior region while a blood vessel is in the posterior region. Molecularly, the ETS-type transcription factor *polyomavirus enhancer activator 3* is expressed in the anterior mesenchyme and in the posterior epithelium [79]. These data suggest that pharyngeal arches are segmented.

Despite their segmental characteristics, rhombomeres and pharyngeal arches are often not considered in comparisons of segmentation among the arthropods, annelids, and chordates. Perhaps this is because rhombomeres and the neural crest component of pharyngeal arches are vertebrate innovations, and therefore do not have homologous counterparts in the arthropods and annelids. Although, as some authors suggest, these seemingly vertebrate specific segmental head structures could have been superimposed upon an ancestral segmental body plan, similar to that of the arthropods and annelids [63] (see [67] for a persuasive counterpoint). Most importantly, the a-p pattern within each rhombomere and pharyngeal arch suggest that they are segmental and therefore should be considered in further studies and discussions of segmentation. As vertebrate innovations, they are especially interesting as models of segmental evolution in novel structures.

## Conclusions

Ideally, a precise definition of segmentation would facilitate our understanding of mechanisms of development, and inform our thoughts on evolutionary processes and events. Instead, more than two millennia of studying segmentation in animals have failed to produce a definition of segmentation that is applicable in even a majority of cases. Moreover, discussions on segmentation are often reduced to debates over the definition of segmentation and whether the animal or system described is actually segmented, rather than to debates over the developmental mechanisms and evolutionary processes.

To bring the focus back to developmental and evolutionary biology, we suggest that ‘segmentation’ be used in its most general meaning: the repetition of units along the a-p axis, where each unit has a-p polarity [20,45]. This inclusive definition should then be combined with an exact description of the segmented structures in the animals or systems being discussed, as well as a clearly stated hypothesis concerning the specific nature of the potential homology of structures. While we support a general definition of segmentation, it is also crucial that authors be explicit in what they are implying about ancestors and shared traits versus convergence, to facilitate the advancement of new ideas, versus circular discussions.

A broader definition of segmentation could also impact molecular studies. New studies on segmentation should examine multiple genes, since there is a wide range of molecular mechanisms involved in segmentation, even within groups with segmental homology. Advancements in sequencing technologies have made it possible to find and analyze many genes involved in the segmentation process simultaneously. Moreover, these studies can yield an unbiased list of genes involved in segmentation in a particular organism or structure, as opposed to the candidate-gene approach used in the past. It will be exciting to see how broader knowledge about segmental molecular mechanisms impacts our thoughts on the core features of segmentation and the shared homology among segmented animals.

## Abbreviations

a-p: anterior-posterior; En: Engrailed; Ph-hh: *Parhyale-hedgehog*; PSPR: Parasegment precursor row.

## Competing interests

The authors declare that they have no competing interests. No funding was received for this review.

## Authors’ contributions

RLH drafted the manuscript. NHP critically revised the manuscript. RLH and NHP read and approved the final manuscript.
